# All-cause and cause-specific mortality following non-traumatic spinal cord injury: evidence from a population-based cohort study in Switzerland

**DOI:** 10.1038/s41393-019-0361-6

**Published:** 2019-10-07

**Authors:** A. Buzzell, J. D. Chamberlain, I. Eriks-Hoogland, K. Hug, X. Jordan, M. Schubert, M. Zwahlen, M. W. G. Brinkhof, for the SwiSCI study group and the Swiss National Cohort

**Affiliations:** 1 Swiss Paraplegic Research, Nottwil, Switzerland; 2 Department of Health Sciences and Medicine, University of Lucerne, Lucerne, Switzerland; 3 Institute of Social and Preventative Medicine, University of Bern, Bern, Switzerland; 4 Swiss Paraplegic Centre, Nottwil, Switzerland; 5 REHAB Basel, Basel, Switzerland; 6 Clinique Romand de Réadaption, Sion, Switzerland; 7 Spinal Cord Injury Center, Balgrist University Hospital, Zurich, Switzerland

**Keywords:** Epidemiology, Epidemiology, Risk factors

## Abstract

**Study design:**

Observational cohort study.

**Objective:**

To benchmark all-cause and cause-specific mortality following NTSCI to the general population (GP).

**Setting:**

Specialized rehabilitation centers in Switzerland.

**Methods:**

Longitudinal data from the Swiss Spinal Cord Injury (SwiSCI) Medical Record study were probabilistically linked with cause of death (CoD) information from the Swiss National Cohort. Standardized mortality ratios (SMRs) were estimated for all-cause and cause-specific mortality. Competing risk frameworks were used to estimate the probability of death due to specific CoD.

**Results:**

One thousand five hundred and one individuals were admitted for first rehabilitation with NTSCI between 1990–2011; CoD information was available for 454 individuals of the 525 individuals that died. Overall, the mortality rate for persons with NTSCI was 1.6 times greater than that of the GP. Deaths due to cardiovascular disease (39.8%), neoplasms (22%), and infection (9.9%) were most often reported. Individuals with an SCI due to a vascular etiology indicated the greatest burden of mortality from infection compared with the GP (SMR 5.4; 95% CI, 3.1 to 9.2).

**Conclusions:**

Cause-specific SMRs varied according to etiology. This supports the need for targeted clinical care and follow-up. Cardiovascular disease, neoplasms, and infection, emerged as main causes of death following NTSCI and should thus be targets for future research and differential clinical management approaches.

## Introduction

The global incidence of non-traumatic spinal cord injuries (NTSCIs) has seen a marked increase in recent years, and this is forecasted to continue given a globally aging population [1]. Although epidemiological evidence for NTSCI is limited, the WHO recently summarized a report which estimated the worldwide incidence to vary between 12 and 76 per million population [2]. However, critical epidemiological indicators, instrumental to informing long-term patient management and health policy—including mortality—remain scarce for NTSCI [3]. While most of the currently available literature focuses on traumatic SCI (TSCI) [4, 5], this evidence is often not transposable to NTSCI due to the distinct, often age-related pathologies associated with NTSCI. This lack of evidence renders evidence-based clinical decisions, prognosis, patient education, and resource allocation for the NTSCI population particularly challenging [3, 6, 7].

Considering the etiologically heterogeneous and elderly composition of the NTSCI population [1, 3], identification of subgroups suitable for targeted interventions are essential for minimizing within-group mortality differentials and improvement of the overall health system [8]. All-cause and cause-specific mortality estimates allow for the investigation of the burden of mortality associated to NTSCI by highlighting risk factors that contribute to an increased risk of mortality and specific cause of death (CoD). In addition, benchmarking the mortality burden in NTSCI with the general population (GP) could facilitate improvement in health system’s resource allocation and models of care through the identification of differences in mortality and CoD relative to NTSCI characteristics and etiology. Therefore, the objective of this study is to estimate the relative risk of all-cause and cause-specific mortality in NTSCI with reference to the GP in Switzerland.

## Methods

### Study description

Data for the present study were collected from the Medical Records study of the Swiss Spinal Cord Injury (SwiSCI) cohort study, which includes all SCI-specific rehabilitation centers in Switzerland from 1990 to 2011 [9]. Eligibility for study inclusion was new cases of NTSCI admitted to rehabilitation in individuals of at least 16 years of age. Cases of NTSCI due to congenital disorders (e.g., spina bifida) or progressive disorders, such as multiple sclerosis and Guillain–Barre syndrome were excluded from the SwiSCI study.

### Data management

Data pertaining to CoD following NTSCI from the SwiSCI cohort were linked with the Swiss National Cohort (SNC) using probabilistic record linkage [10]; birthdate, date of death, age, sex, and geocoded address were used for linkage [4]. In total, 85.5% of SwiSCI records were linked, 92.4% of the linked data had no alternative matches. For linkages with multiple potential matches, a linkage weight was used to assess linkage quality. All main analyses included within the present study, used records with the highest weighted linkage; alternative linkages were used in sensitivity analysis to assess the potential influence of the alternative mortality record linkage with CoD-related estimates, particularly for cause-specific standardized mortality ratios (SMRs).

NTSCI etiologies were categorized according to a hierarchical classification strategy described in the “International SCI core data sets for NTSCI” [11] and grouped into the following categories: “degenerative disc disorder,” “infection,” “vascular disorder,” “benign tumor,” and “malignant tumor.” Individuals that developed an NTSCI from an unclear origin, metabolic disorders, inflammatory disease, and radiation-related causes were grouped into an “other” category. Tumor-related NTSCIs without a specified tumor status (i.e., malignant or benign) were excluded from all statistical analyses (*n* = 10). SCI-related characteristics comprised “lesion level” (paraplegia/tetraplegia) and “lesion completeness” (incomplete/complete). A combined variable categorized as “lesion severity” comprised of lesion level and completeness (e.g., paraplegia-incomplete, paraplegia-complete, tetraplegia-incomplete, tetraplegia-complete), according to the “International Spinal Cord Injury core data set” guidelines [12].

Information on cause of death were based on documentation from death certificates obtained from the linkage with the SNC. Cause of death was mainly coded using International classification of diseases (ICD) version 10. 11 cases were originally coded using ICD version 8 and subsequently matched with ICD-10 codes based on name and description (ICD version 9 was never implemented in the Swiss healthcare system). In line with other CoD studies, the underlying cause of death, defined as the disease or injury that initiated the course of events leading directly to death was utilized in all analyses [13, 14]. With the exception of descriptive statistics, CoD was categorized into four main subgroups based on contemporary mortality studies that have implemented death certificate data [4, 14], namely: “cardiovascular disease (CVD),” “infection,” “neoplasm,” and “other.” The CVD category included: death due to cardiac disease, ischemic heart disease, cerebral circulatory disease, pulmonary circulatory disease, as well as other circulatory disease while the neoplasm category included both benign and malignant tumors. The infection disease category comprised deaths due to respiratory infection, urinary infection, infectious disease, or septicemia. The “other” category additionally included all other causes of death including digestive-related diseases. Information on the population distribution of NTSCI etiological groups was presented previously [15].

### Statistical analysis

Descriptive statistics include raw numbers and percentages to evaluate differences in causes of death across major etiological subgroups. To prevent misinterpretation and skewed results, subsequent estimates excluded NTSCIs of malignant origin, as reported CoD pertaining to these cases was almost exclusively due to a neoplasm. Negative binomial regression was used to estimate SMRs and corresponding confidence intervals for all-cause mortality using age-, sex-, and calendar year-specific mortality rates from the Swiss GP as offset. Cumulative incidence over 21 years of follow-up was estimated within a competing risk framework for estimation of cause-specific subhazard ratios (sHRs) using the Fine and Gray model [16].

Multiple imputation via chained equations was used to account for potential bias due to missing information in completeness of lesion and lesion level; data were presumed missing at random. The imputation model included all predictor variables used in regression modeling (e.g., sex, age category, lesion level, lesion completeness, decade of SCI, NTSCI etiology), as well as relevant parameters of the outcome (i.e., vital status indicator [yes/no] and the log of survival time) [17]. Twenty imputed data sets were created to replace missing values [18]; estimates generated from imputed data sets were combined using Rubin’s rules to derive global estimates [19].

All analyses were implemented using Stata software version 14.2 for Windows (College Station, TX). Furthermore, the methodology and results were reported according to the recommendations outlined in the Strengthening the Reporting of Observational Studies in Epidemiology (STROBE) guidelines [20].

## Results

### Participant characteristics

The present study includes 1501 persons diagnosed with an NTSCI between January 1st, 1990 and September 30th, 2011, with a cumulative person-time of 7457 person-years and 525 individuals with a known date of death. Information on cause of death was available for 454 individuals.

### All-cause mortality

The all-cause mortality risk for persons with NTSCI with respect to the GP are shown in Table 1. The overall mortality rate for individuals with NTSCI was estimated to be 1.6 times greater than that of the GP in Switzerland. Individuals in the youngest age category had the highest mortality rate with reference to the GP (SMR = 20.7; 95% CI, 4.1–37.3). Cases of NTSCI due to infection had a mortality rate 2.5 times greater than the GP (95% CI,1.5–3.5), while those with vascular etiology had an SMR of 1.7 (95% CI, 1.3–2.1) (Table 1). Individuals with complete lesions were estimated to have a mortality burden over three times greater than that of the GP (SMR = 3.2; 95% CI, 2.0–4.3), while lesion level was not associated with elevated mortality.

**Table 1:** All-cause SMRs (for all nonmalignant NTSCI etiological groups)

Characteristics	SMR (95% CI)	*P*-value
Overall	1.62 (1.34–1.90)	
Sex		0.18
Male	1.72 (1.39–2.04)	
Female	1.43 (1.08–1.78)	
Age category		<0.001
16–30	20.7 (4.14–37.3)	
31–45	5.33 (2.18–8.48)	
45–60	2.63 (1.74–3.51)	
61–75	1.57 (1.27–1.88)	
75+	1.10 (0.91–1.29)	
Lesion level		0.60
Paraplegia	1.65 (1.34–1.96)	
Tetraplegia	1.53 (1.13–1.94)	
Completeness		<0.001
Incomplete	1.43 (1.18–1.68)	
Complete	3.15 (2.03–4.27)	
Lesion severity		<0.001
Paraplegia, incomplete	1.43 (1.13–1.72)	
Paraplegia, complete	2.98 (1.82–4.13)	
Tetraplegia, incomplete	1.40 (1.01–1.78)	
Tetraplegia, complete	6.18 (0.92–11.44)	
Etiology		<0.001
Degenerative disc disorder	1.10 (0.81–1.39)	
Infection	2.53 (1.53–3.53)	
Vascular disorder	1.73 (1.34–2.12)	
Benign tumor	1.46 (0.86–2.06)	
Other	3.20 (1.71– 4.68)	

*SMR* standardized mortality ratio

### Cause-specific mortality

Table 2 summarizes main causes of death in the cohort over the entire study period stratified by whether malignant or nonmalignant etiology of NTSCI. CVDs were the most commonly reported CoD in nonmalignant NTSCI etiologies (39.8%) followed by neoplasms (21.7%). In addition, digestive-related diseases accounted for 6.3% of deaths following NTSCI. Table S1 presents cause of death stratified by NTSCI etiology. 36.7% of deaths in individuals with an NTSCI due to a degenerative disc disorder were due to CVD, while 22% of deaths in this group were related to neoplasms. Furthermore, cause of death in individuals with an NTSCI due to malignant tumors were almost exclusively related to neoplasms (Table S2). Therefore cancer-specific causes of death in individuals with NTSCI due to a malignant neoplasm are additionally presented in Table S2. 39.5% of deaths due to neoplasms were specifically from a sex-specific neoplasm, followed by “neoplasms of lymphoid hematopoietic tissue” at 19.7%.

**Table 2:** Cause of death stratified by NTSCI etiology: nonmalignant etiology compared with malignant tumor

	Nonmalignant etiologies^a^	Malignant tumor
Cause of death (ICD-10 code)	*n* (%)	*n* (%)
Respiratory infection (J00-J22)	12 (4.4)	1 (0.5)
Chronic obstructive pulmonary disease (J40–J47)	7 (2.6)	0 (0.0)
Other respiratory disease (J30–J99)	7 (2.6)	0 (0.0)
Cardiac disease (I05–I09, I11; I30–I59)	20 (7.4)	2 (1.1)
Ischemic heart disease (I20–I25)	34 (12.5)	1 (0.5)
Cerebral, circulatory disease (I60–I69)	19 (7.0)	2 (1.1)
Pulmonary, circulatory disease (I26–I28)	2 (0.7)	0 (0.0)
Other circulatory disease (I10; I12–I15; I70–I99)	19 (7.0)	1 (0.5)
Neoplasms (C00-D49)	59 (21.7)	157 (87.7)
Urinary infection (N30–N39)	4 (1.5)	0 (0.0)
Renal failure (N17–N19)	3 (1.1)	0 (0.0)
Other urogenital (N00–N16, N20; N40–N99)	2 (0.7)	0 (0.0)
Digestive-related disease (K00–K95)	17 (6.3)	1 (0.5)
Suicide (X71–X83)	4 (1.5)	4 (2.2)
Accidents (S00-T88; V00-X58)	5 (1.8)	0 (0.0)
Skin-related disease (L00–L99)	1 (0.4)	0 (0.0)
Infectious disease (A00-B99, excl. A41)	6 (2.2)	2 (1.1)
Septicemia (A41)	5 (1.8)	0 (0.0)
Ill-defined (R00-R99)	8 (2.9)	3 (1.6)
Nervous system-related disease (G00–G99)	15 (5.5)	0 (0.0)
Endocrine-related disease (E00–E89)	10 (3.7)	2 (1.1)
Musculoskeletal-related disease (M00–M99)	8 (2.9)	2 (1.1)
Mental-related disease (F01–F99)	4 (1.5)	0 (0.0)
Immune-, blood-, eye/ear-related disease (D50–D89; H00–H59)	1 (0.4)	1 (0.5)
Total	272 (100)	179 (100)

^a^Nonmalignant etiological groups include: degenerative disc disorder, infection, vascular disorder, benign tumors, and other

### Differential mortality

Cause-specific mortality rates according to NTSCI etiology and lesion completeness are presented in Table 3. Compared with the general population, individuals with an NTSCI due to a degenerative disc disorder were over three times more likely to die of infection (SMR = 3.2; 95% CI, 1.7–5.9), (Table 3). In addition, persons with degenerative disc disorders exhibited elevated mortality rates from CVD 1.6 times greater than that of the GP (95% CI, 1.1–2.3). Persons with an NTSCI originating from a vascular disorder experienced an elevated risk of mortality due to CVD (SMR = 4.1; 95% CI, 3.2–5.3). However, the greatest burden of mortality identified in this vascular etiological group was due to infection (SMR = 5.4; 95% CI, 3.1–9.2). For individuals with an infection-related NTSCI, death due to infection was elevated with respect to the general population (SMR = 4.4; 95% CI, 1.4–13.5) (Table 3). Cause-specific SMRs were elevated across most etiological groups in individuals with a complete lesion. For example, among individuals with a vascular-related NTSCI, infection-related SMRs were roughly double for complete lesions in comparison with incomplete lesions (SMR = 9.6; 95% CI, 4.0–23.0 and SMR = 4.32; 95% CI, 2.3–8.3, respectively) (Table 3). Similar SMRs were estimated for the respective cause of death group according to NTSCI etiology when using alternative linkages (Table S4).

**Table 3:** Cause-specific SMRs stratified by NTSCI etiological groups

	Cause of death^a^			
	CVD	Infection^b^	Neoplasm	Other^c^
NTSCI etiology				
Degenerative disc disorder				
Total	1.62 (1.13–2.32)	3.19 (1.72–5.92)	1.45 (0.91–2.30)	1.52 (0.94–2.44)
Incomplete lesion	1.53 (1.05–2.21)	3.22 (1.73–5.99)	1.47 (0.93–2.34)	1.54 (0.96–2.47)
Complete lesion	3.88 (1.46–10.35)	5.55 (0.78–39.38)	2.31 (0.58–9.24)	4.22 (1.36–13.08
Infection etiology				
Total	1.49 (0.67–3.33)	4.36 (1.41–13.51)	2.16 (0.97–4.81)	6.09 (3.67–10.11)
Incomplete lesion	1.13 (0.42–3.02)	3.31 (0.83–13.24)	1.65 (0.62–4.39)	5.03 (2.79–9.09)
Complete lesion	4.92 (1.85–13.10)	7.13 (1.00–50.62)	3.62 (0.90–14.47)	8.30 (3.12–22.13)
Vascular disorder				
Total	4.12 (3.20–5.32)	5.36 (3.11–9.22)	1.45 (0.88–2.41)	2.06 (1.30–3.26)
Incomplete lesion	3.51 (2.60–4.73)	4.32 (2.25–8.31)	1.59 (0.94–2.68)	1.86 (1.10–3.13)
Complete lesion	6.07 (3.87–9.51)	9.55 (3.97–22.94)	0.47 (0.07–3.32)	3.93 (1.87–8.25)
Benign tumor				
Total	0.51 (0.16–1.58)	–	3.77 (2.35–6.07)	–
Incomplete lesion	–	–	3.63 (2.22–5.92)	–
Complete lesion	–	–	4.23 (1.06–16.93)	–
Other etiology				
Total	4.18 (2.25–7.77)	–	1.60 (0.52–4.95)	7.31 (3.93–13.58)
Incomplete lesion	4.64 (2.42–8.92)	–	1.89 (0.61–5.86)	7.10 (3.55–14.19)
Complete lesion	3.43 (0.86–13.72)	–	2.69 (0.38–19.12)	9.88 (3.19–30.62)

*CVD* cardiovascular diseases

^a^Cause of death are grouped into the most common categories

^b^The CoD “Infection” includes respiratory infections, urinary infections, septicemia, in addition to all other infectious disease (A00-B99)

^c^All other causes of death are grouped into the “other” category

### Competing risks

sHRs for competing risks of mortality are presented in Table 4. Age was a prominent risk factor for all major causes of death. An elevated risk of mortality associated with the original NTSCI etiology was apparent within each CoD category. For example, the sHR for mortality due to CVD was elevated for vascular-related NTSCIs (sHR = 1.80; 95% CI, 1.01–3.11). Similarly, for mortality due to neoplasms, a greater sHR was estimated for individuals with a benign tumor NTSCI etiology (sHR 4.0; 95% CI, 1.8–8.6).

**Table 4:** Competing risk analysis of risk factors for cause-specific mortality

	Cause of death					
	Cardiovascular diseases (*n* = 108)		Infection (*n* = 27)		Neoplasms (*n* = 59)	
Characteristics	sHR (95% CI)	*P-*value	sHR (95% CI)	*P-*value	sHR (95% CI)	*P-*value
Age at injury						
<60 years	Ref.	<0.0001	Ref.	0.09	Ref.	0.05
60–75 years	3.85 (2.00–7.41)		3.33 (1.11–9.99)		2.11 (1.10–4.04)	
75+ years	8.26 (4.20–16.23)		2.74 (0.80–9.34)		2.18 (0.99–4.78)	
Lesion level						
Paraplegia	Ref.	0.44	Ref.	0.77	Ref.	0.87
Tetraplegia	0.81 (0.48–1.38)		1.15 (0.45–2.90)		1.05 (0.57–1.97)	
Completeness						
Incomplete	Ref.	0.11	Ref.	0.05	Ref.	0.58
Complete	1.65 (0.90–3.03)		2.82 (1.00–8.01)		0.74 (0.25–2.17)	
NTSCI etiology						
Degenerative disc disorder	Ref.	0.01	Ref.	0.82	Ref.	<0.01
Infection	0.58 (0.20–1.66)		1.21 (0.31–4.65)		1.55 (0.59–4.09)	
Vascular disorder	1.80 (1.04–3.11)		1.14 (0.43–3.07)		1.10 (0.50–2.43)	
Benign tumor	0.39 (0.12–1.29)		0.41 (0.05–3.23)		3.98 (1.84–8.64)	

*sHR* subhazard ratio

## Discussion

### All-cause and cause-specific mortality

Compared with the general population, mortality rates were 1.6 times greater in the NTSCI population. Cause-specific SMRs varied greatly between etiological groups and according to lesion completeness. A particularly vulnerable group identified in the present study were individuals with an NTSCI due to an infection, who experienced a greater burden of infection-related mortality. These results indicate that individuals with infection pathology potentially sustain an immunodeficiency beyond treatment for NTSCI, which may contribute to their mortality following injury. Furthermore, individuals with an NTSCI due to a vascular disorder had a greater risk of mortality due to a CVD, suggesting that underlying health conditions resulting in NTSCI play a critical role in the mortality in addition to the spinal cord lesion [21].

### Differential mortality

Estimates from this study highlight the potential influence of NTSCI etiology on differential causes of death. For example, deaths due to CVDs were most common in this population and may be attributable to treatable health conditions, such as hypertension, obesity, or dyslipidemia [22]. Injury-related dysreflexia and orthostatic hypotension may further contribute to reduced life expectancy due to CVD [23]. Our results are supported by findings from a population-based community survey that looked at perceived health conditions following SCI in Switzerland; 44% of participants with NTSCI reported circulatory problems, and over 25% experienced autonomic dysreflexia [24]. In addition, the burden of mortality due to digestive-related diseases was greater in the NTSCI population compared with the GP. Competing risk analyses investigating risk of mortality due to digestive-related diseases was not possible given the diverse categorization of CoDs in this group which includes alcoholic cirrhosis of the liver, perforation of the intestine, as well as duodenal ulcers. However, persons with NTSCI were found to have a nearly 11 times higher rate of mortality from a digestive-related CoD (SMR 10.9 95% CI, 6.8–17.5) in comparison with the GP (Table S3). This enhanced burden of mortality experienced by the NTSCI population for digestive-related CoDs may be partly due to the multimorbid nature of NTSCI, related to a predisposition to gastrointestinal disorders in addition; obesity and alcoholism may further enhance the burden within this community [25, 26].

### Personalized rehabilitation and long-term follow-up programs

Long-term follow-up care and etiology-specific, tailored treatments could potentially reduce complications from secondary health conditions and improve survival outcomes. A recent study found that individuals with a nonmalignant NTSCI can expect ~10–12 life years remaining with an attained age of 60 years, underscoring the importance of long-term follow-up care for this population [15]. In addition, patient education that enables individuals with NTSCI and their caretakers to effectively seek care and take care in preventative methods (e.g., low-impact exercise, dietary intervention, and pressure sore prevention) could potentially minimize the development of secondary complications and lower the risk of mortality and improve quality of life [27].

Healthcare systems and NTSCI management models must consider the differential prognoses and mortality outcomes identified between NTSCI etiological groups to aid in the development of specialized inpatient and outpatient rehabilitation programs. For example, individuals with progressive forms of NTSCI (e.g., malignant and benign tumors) can benefit from short-term rehabilitation plans, with optimized goals, aimed at improving vital aspects of functioning (e.g., bladder management and caretaker education) [28–30]. Individuals with an infection and cardiovascular etiology additionally require intensive routine checks and up-to-date vaccinations to minimize medical complications that can interfere with rehabilitation [31]. Specialized outpatient follow-up care can play a vital role for the prevention and treatment of complications secondary to NTSCI [29]. Outpatient services, such as the Swiss-based ParaHelp [32], can provide individuals with NTSCI with quality advice and counseling and reduce patient burden associated with in-clinic follow-up. Specialized healthcare approaches are needed for the successful management of NTSCIs across the disease continuum, from initial diagnosis to long-term community care and support, to facilitate improved health outcomes and reduce etiology-specific disparities.

### Strengths and limitations

A major strength of this study is that it used data from a multicenter rehabilitation cohort, providing a clear sampling frame of people who were admitted to first rehabilitation in Switzerland over a 21-year study period. Furthermore, stratification of NTSCI etiological groups for all analyses allows for comparison of mortality outcomes between groups. This study is one of the first population-based studies of relative mortality following NTSCI.

The study design targeted individuals that were admitted for first rehabilitation, therefore the portion of individuals that never receive specialized rehabilitation or treatment are less represented [3, 5], which can limit the generalizability of results to the entire population with NTSCI. Thus, SMRs of individuals not admitted to specialized care may be even higher. Another potential limitation is that information on cause of death relied solely on the accuracy of reporting practices on death certificates. However, the uncertainty regarding the reporting of CoD would likely have been comparable in the general population, resulting in minimal bias due to differential mortality in CoD coding [33]. Although there is no official protocol to identify analogous codes between ICD 8 and ICD 10, this study only includes 11 cases coded using ICD version 8, therefore we do not expect a large impact on results. There is additional uncertainty related to linkage reliability and quality [33]. However, this is unlikely to be differential, and would therefore merely impact precision [4, 33]. Sensitivity analyses that checked alternative links did not identify meaningful differences (Table S4). Many CoD groups included limited cases, which were further reduced when stratified on NTSCI-specific etiological groups. Thus, caution is required when drawing conclusions from SMRs in groups with small sample sizes. Collaborative efforts in NTSCI with respect to mortality outcomes in high income settings are needed to improve statistical power, potentially in the form of multinational studies with longer follow-up time to improve inference. Finally, linkage with cancer registries could provide a basis for mortality comparisons in individuals with NTSCI from a neoplastic origin.

## Conclusion

Cause-specific mortality differentials identified between NTSCI etiological groups indicate a need for specialized rehabilitation strategies that align NTSCI-specific treatment with care for underlying health conditions. Furthermore, research involving neurorehabilitation and healthcare strategies following NTSCI that includes initiatives for reducing risk of CVD, infections, and gastrointestinal dysfunction is needed to optimize morbidity and mortality outcomes.

## Supplementary information

Supplementary Tables

## Data Availability

Owing to our commitment to SwiSCI study participants and their privacy, data sets generated during the current study are not made publicly available. The SwiSCI study center requires, on behalf of the SwiSCI Study Group, contact prior to any planned data usage (contact@swisci.ch).
