# Supplementary material for: All-cause and cause-specific mortality following non-traumatic spinal cord injury: evidence from a population-based cohort study in Switzerland
Source: Spinal Cord. 2019 Oct 7;58(2):157–64. doi: 10.1038/s41393-019-0361-6 (PMC7007408; doi:10.1038/s41393-019-0361-6)
Supplement: Supplementary file 1 — Supplementary Tables [file 41393_2019_361_MOESM1_ESM.pdf]

Buzzell A, Chamberlain JD, Eriks-Hoogland I, Hug K, Jordan X, Schubert M, Zwahlen M, Brinkhof MWG, for the SwiSCI study group and the Swiss National Cohort, All-cause and cause-specific mortality following non-traumatic spinal cord injury: evidence from a population-based cohort study in Switzerland (2019)

Supplementary information:

Table S1: Cause of death stratified by NTSCI-specific etiologies

This table provides information on underlying cause of death across NTSCI-specific etiologies.

Table S2: Cancer-specific causes of death within individuals with an NTSCI from a neoplastic etiology

This table provides information on the cancer-specific causes of death in individuals with NTSCI originating from a malignant tumor.

Table S3: Cause-specific SMRs within all non-malignant cases of NTSCI

This table provides supplementary information on cause-specific SMRs for all non-malignant cases of NTSCI (main results Table 3).

Table S4: Cause-specific SMRs using alternative linkage

This table provides a sensitivity analysis of SMRs from the alternative record linkages.

Supplementary Table 1: Cause of death according to NTSCI etiologies

	**Degenerative disc disorder**	**Infection**	**Vascular**	**Other**	**Benign tumor**	**Malignant tumor**
Cause of death (ICD-10 code)	n (%)	n (%)	n (%)	n (%)	n (%)	n (%)
Respiratory infection (J00-J22)	4 (4.9)	1 (3.1)	6 (5.5)	0 (0.0)	1 (4.2)	1 (0.6)
Chronic obstructive pulmonary disease (J40-J47)	3 (3.7)	0 (0.0)	3 (2.8)	1 (4.0)	0 (0.0)	0 (0.0)
Other respiratory disease (J30-J99)	2 (2.4)	0 (0.0)	4 (3.7)	1 (4.0)	0 (0.0)	0 (0.0)
Cardiac disease (I05-I09, I11; I30-I59)	5 (6.1)	1 (3.1)	10 (9.2)	4 (16.0)	0 (0.0)	2 (1.1)
Ischemic heart disease (I20-I25)	13 (15.9)	3 (9.4)	16 (14.7)	1 (4.0)	1 (4.2)	1 (0.5)
Cerebral, circulatory disease (I60-I69)	4 (4.9)	0 (0.0)	13 (11.9)	1 (4.0)	1 (4.2)	2 (1.1)
Pulmonary, circulatory disease (I26-I28)	0 (0.0)	1 (3.1)	1 (0.9)	0 (0.0)	0 (0.0)	0 (0.0)
Other circulatory disease (I10; I12-I15; I70-I99)	3 (3.7)	1 (3.1)	12 (11.0)	2 (8.0)	1 (4.2)	1 (0.5)
Neoplasms (C00-D49)	18 (22.0)	6 (18.8)	15 (13.8)	3 (12.0)	17 (70.8)	157 (87.7)
Urinary infection (N30-N39)	0 (0.0)	1 (3.1)	3 (2.8)	0 (0.0)	0 (0.0)	0 (0.0)
Renal failure (N17-N19)	0 (0.0)	0 (0.0)	3 (2.8)	0 (0.0)	0 (0.0)	0 (0.0)
Other urogenital (N00-N16, N20; N40-N99)	1 (1.2)	0 (0.0)	1 (0.9)	0 (0.0)	0 (0.0)	0 (0.0)
Digestive-related disease (K00-K95)	7 (8.5)	2 (6.3)	4 (3.7)	2 (8.0)	2 (8.3)	1 (0.5)
Suicide (X71-X83)	0 (0.0)	3 (9.4)	0 (0.0)	0 (0.0)	1 (4.2)	4 (2.2)
Accidents (S00-T88; V00-X58)	2 (2.4)	1 (3.1)	2 (1.8)	0 (0.0)	0 (0.0)	0 (0.0)
Skin-related disease (L00-L99)	1 (1.2)	0 (0.0)	0 (0.0)	0 (0.0)	0 (0.0)	0 (0.0)
Infectious disease (A00-B99, excl. A41)	3 (3.7)	1 (3.1)	2 (1.8)	0 (0.0)	0 (0.0)	2 (1.1)
Septicemia (A41)	3 (3.7)	0 (0.0)	2 (1.8)	0 (0.0)	0 (0.0)	0 (0.0)
Ill-defined (R00-R99)	3 (3.7)	1 (3.1)	4 (3.7)	0 (0.0)	0 (0.0)	3 (1.6)
Nervous system related disease (G00-G99)	2 (2.4)	5 (15.6)	6 (5.5)	2 (8.0)	0 (0.0)	0 (0.0)
Endocrine-related disease (E00-E89)	1 (1.2)	4 (12.5)	2 (1.8)	3 (12.0)	0 (0.0)	2 (1.1)
Musculoskeletal related disease (M00-M99)	5 (6.1)	1 (3.1)	0 (0.0)	2 (8.0)	0 (0.0)	2 (1.1)
Mental-related disease (F01-F99)	2 (2.4)	0 (0.0)	0 (0.0)	2 (8.0)	0 (0.0)	0 (0.0)
Immune, blood, eye/ear related disease (D50-D89; H00-H59)	0 (0.0)	0 (0.0)	0 (0.0)	1 (4.0)	0 (0.0)	1 (0.5)
Total	82 (100)	32 (100)	109 (100)	25 (100)	24 (100)	179 (100)

Supplementary Table 2: Cancer-specific cause of death within individuals having an NTSCI originating from a malignant tumor (n=157)[1]

Cause of death (ICD-10 code)	Female	Male	Total
**Malignant Neoplasms (C00-C97)**			
Oral neoplasm (C00-C14)	0 (0.0)	1 (0.9)	1 (0.6)
Digestive neoplasm (C15-C26)	4 (8.5)	5 (4.6)	9 (5.7)
Respiratory neoplasm (C30-C39)	4 (8.5)	12 (10.9)	16 (10.2)
Melanomas of the skin/soft tissue (C43-C49)	2 (4.3)	6 (5.5)	8 (5.1)
Sex-specific neoplasm[2]: (C50-C63)	20 (42.6)	42 (38.2)	62 (39.5)
Urinary neoplasm (C64-C68)	3 (6.4)	8 (7.3)	11 (7.0)
Neoplasm of the eye, brain, CNS (C69-C72)	4 (8.5)	5 (4.6)	9 (5.7)
Ill-defined neoplasms (C76-C78)	2 (4.3)	8 (7.3)	10 (6.4)
Lymphoid/hematopoietic tissue (C81-C96)	8 (17.0)	23 (20.9)	31 (19.7)
Total	47 (100.0)	110 (100.0)	157 (100.0)

[1]Only including individuals with an NTSCI due to a malignant tumor

[2]Sex-specific neoplasms in females included neoplasms of the breast (n=18) and neoplasms of the genital organs (n=2). Sex-specific neoplasms in males were related to genital organs (n=42).

Supplementary Table 3: Cause-specific SMRs within all non-malignant cases of NTSCI

Cause of death (ICD-10 code)	Number of deaths	Expected deaths	SMRs	95% CI
Respiratory infection (J00-J22)	12	3.10	3.87	2.20-6.82
Chronic obstructive pulmonary disease (J40-J47)	7	4.03	1.74	0.83-3.65
Other respiratory disease (J30-J99)	7	5.43	1.29	0.61-2.70
Cardiac disease (I05-I09; I11; I30-I59)	20	10.33	1.94	1.25-3.00
Ischemic heart disease (I20-I25)	34	18.75	1.81	1.30-2.54
Cerebral, circulatory disease (I60-I69)	19	8.08	2.35	1.50-3.69
Pulmonary, circulatory disease (I26-I28)	2	0.76	2.62	0.66-10.48
Other circulatory disease (I10; I12-I15; I70-I99)	19	7.17	2.65	1.69-4.15
Neoplasms (C00-D49)	59	31.88	1.85	1.43-2.39
Urinary infection (N30-N39)	4	0.42	9.62	3.61-25.62
Renal failure (N17-N19)	3	0.71	4.21	1.36-13.05
Other urogenital (N00-N16, N20; N40-N99)	2	4.46	0.45	0.11-1.79
Digestive-related disease (K00-K95)	17	1.56	10.87	6.76-17.49
Suicide (X71-X83)	4	3.65	1.10	0.41-2.92
Accidents (S00-T88; V00-X58)	5	0.16	31.44	13.08-75.53
Skin-related disease (L00-L99)	1	1.17	0.85	0.12-6.05
Infectious disease (A00-B99, excl. A41)	6	0.34	17.49	7.86-38.92
Septicemia (A41)	5	3.83	1.31	0.54-3.14
Ill-defined (R00-R99)	8	5.45	1.47	0.73-2.94
Nervous system related disease (G00-G99)	15	3.35	4.48	2.70-7.44
Endocrine-related disease (E00-E89)	10	0.89	11.28	6.07-20.97
Musculoskeletal related disease (M00-M99)	8	5.85	1.37	0.68-2.73
Mental-related disease (F01-F99)	4	0.31	12.98	4.87-34.58
Immune, blood, eye/ear related disease (D50-D89; H00-H59)	1	1.55	0.64	0.09-4.57

This table provides supplementary information on cause-specific SMRs for all non-malignant cases of NTSCI (main results Table 3).

Supplementary Table 4: Cause-specific SMRs (95% CI) according to NTSCI etiology using the alternative record linkage

	Cause of death[1]			
	CVD	Infection[2]	Neoplasm	Other[3]
**NTSCI Etiology**				
Degenerative disc disorder				
Total	1.38 (0.94-2.05)	2.94 (1.53-5.65)	1.40 (0.87-2.26)	1.93 (1.26-2.96)
Incomplete lesion	1.29 (0.85-1.94)	2.97 (1.55-5.71)	1.43 (0.89-2.30)	1.95 (1.27-3.00)
Complete lesion	3.94 (1.48-10.5)	5.66 (0.80-40.2)	1.20 (0.17-8.52)	4.32 (1.39-13.4)
Infection				
Total	1.49 (0.67-3.33)	4.36 (1.41-13.52)	1.80 (0.75-4.33)	6.10 (3.68-10.1)
Incomplete lesion	1.13 (0.42-3.02)	3.31 (0.83-13.24)	1.65 (0.62-4.39)	5.03 (2.79-9.09)
Complete lesion	4.92 (1.85-13.1)	7.15 (1.01-50.8)	1.82 (0.26-12.9)	8.34 (3.13-22.2)
Vascular disorder				
Total	3.69 (2.81-4.84)	5.43 (3.15-9.35)	1.47 (0.89-2.44)	2.89 (1.95-4.28)
Incomplete lesion	3.32 (2.43-4.52)	4.39 (2.28-8.44)	1.61 (0.95-2.71)	2.29 (1.42-3.68)
Complete lesion	4.79 (2.89-7.95)	9.55 (3.97-22.9)	0.47 (0.07-3.32)	6.18 (3.42-11.2)
Benign tumor				
Total	-	-	3.55 (2.18-5.80)	-
Incomplete lesion	0.51 (0.17-1.59)	-	3.40 (2.05-5.64)	-
Complete lesion	-	-	4.26 (1.06-17.0)	-
Other etiology				
Total	3.45 (1.73-6.90)	-	1.62 (0.52-5.02)	8.97 (5.10-15.8)
Incomplete lesion	3.75 (1.79-7.87)	-	1.92 (0.62-5.95)	9.12 (4.91-17.0)
Complete lesion	3.43 (0.86-13.7)	-	2.69 (0.38-19.1)	9.88 (3.19-30.6)

This table provides a sensitivity analysis of SMRs from the alternative record linkages.

CVD=Cardiovascular disease. [1]Cause of death (CoD) is grouped into four the most common categories. [2]The CoD ‘Infection’ includes respiratory infections, urinary infections, septicemia, in addition to all other infectious disease (A00-B99). [3]All other CoD’s are grouped into the “Other” category.
